# Estimated Glomerular Filtration Rate Variability in Patients with Diabetes Receiving SGLT2 Inhibitors Versus DPP4 Inhibitors

**DOI:** 10.3390/pharmaceutics17111370

**Published:** 2025-10-23

**Authors:** Yi-Wei Kao, Tze-Fan Chao, Yu-Wen Cheng, Shao-Wei Chen, Yi-Hsin Chan

**Affiliations:** 1 Department of Financial Technology Applications, Ming Chuan University, Taoyuan 333, Taiwan; 2 Department of Applied Statistics and Information Science, Ming Chuan University, Taoyuan 242, Taiwan; 3 Division of Cardiology, Department of Medicine, Taipei Veterans General Hospital, Taipei 112, Taiwan; 4 Institute of Clinical Medicine, Cardiovascular Research Center, National Yang Ming Chiao Tung University, Taipei 112, Taiwan; 5 The Cardiovascular Department, Chang Gung Memorial Hospital, Linkou, Taoyuan 333, Taiwan; 6 College of Medicine, Chang Gung University, Taoyuan 333, Taiwan; 7 Division of Thoracic and Cardiovascular Surgery, Department of Surgery, Chang Gung Memorial Hospital, Linkou Medical Center, Chang Gung University, Taoyuan 333, Taiwan; 8 Center for Big Data Analytics and Statistics, Chang Gung Memorial Hospital, Taoyuan 333, Taiwan; 9 School of Traditional Chinese Medicine, College of Medicine, Chang-Gung University, Taoyuan 33302, Taiwan; 10 Microscopy Core Laboratory, Chang Gung Memorial Hospital, Linkou, Taoyuan 333, Taiwan

**Keywords:** eGFR variability, sodium–glucose cotransporter 2 inhibitor, dipeptidyl peptidase-4 inhibitor, major adverse kidney event, type 2 diabetes

## Abstract

**Background:** Major clinical trials regarding sodium–glucose cotransporter 2 inhibitors (SGLT2is) have focused on estimated glomerular filtration rate (eGFR) slope and composite kidney outcomes, with limited evaluation of eGFR variability as a kidney outcome or its long-term implications in patients receiving SGLT2i versus placebo. **Methods:** This retrospective study analyzed 3777 propensity score-matched patients with type 2 diabetes receiving either SGLT2i or dipeptidyl peptidase-4 inhibitor (DPP4i) between June 2016 and December 2021. Each patient had eGFR data at three time points before (−15, −9, and −3 months) and after (3, 9, and 15 months) the drug-index date. eGFR variability was assessed using the coefficient of variation (COV) and standard deviation (SD) of eGFR values. **Results:** SGLT2i therapy was associated with a significant reduction in eGFR variability by both COV (from 0.072 (0.001) to 0.069 (0.001); *p* = 0.014) and SD (mL/min/1.73 m^2^) (from 5.34 (0.07) to 4.82 (0.07); *p* < 0.001). In contrast, DPP4i therapy resulted in increased COV (from 0.072 (0.001) to 0.080 (0.001); *p* < 0.001) and no significant improvement in SD (from 5.06 (0.07) to 5.22 (0.07); *p* = 0.082). Greater reduction in eGFR variability was observed in SGLT2i relative to DPP4i with high pre-treatment eGFR variability, pre-existing chronic kidney disease, or rapid pre-treatment eGFR decline. Greater pre-treatment eGFR variability predicted risk of major adverse kidney events (MAKEs) and abrupt kidney decline in DPP4i-treated patients, but not in those on SGLT2is. SGLT2i consistently reduced the risk of MAKE and abrupt kidney decline across the spectrum of pre-treatment eGFR variability, with a greater risk reduction on the MAKE for SGLT2i versus DPP4i therapy with a higher pre-treatment eGFR variability estimated by SD (*p* interaction = 0.014). **Conclusions:** SGLT2i therapy improved eGFR variability and reduced the risk of adverse kidney outcomes compared to DPP4i, particularly in patients with higher pre-treatment eGFR variability.

## 1. Introduction

Type 2 diabetes (T2D) is a major global health concern, significantly increasing the risk of macrovascular or microvascular complications and diabetic kidney disease (DKD) [1]. People with DKD exhibit a substantially faster decline in estimated glomerular filtration rate (eGFR), with mean decline slopes ranging from 1.5 to 4.0 mL/min/1.73 m^2^ per year [2]. While the serial eGFR values and chronic slope have been frequently estimated for assessing kidney function decline and predicting the progression of chronic kidney disease (CKD) and end-stage kidney disease (ESKD) [3,4,5], emerging evidence suggests that eGFR variability—defined as the fluctuation of eGFR values between serial measurements [6]—may offer additional, clinically significant insights. Recent studies have revealed that greater eGFR variability is independently associated with increased risks of ESKD, cardiovascular events, and all-cause mortality, regardless of the underlying chronic eGFR slope [6,7,8,9,10,11,12]. Notably, even individuals with stable eGFR slopes may still experience poor outcomes if their eGFR values fluctuate markedly over time. Sodium–glucose cotransporter 2 inhibitors (SGLT2is) have been shown to be an effective therapy for individuals with DKD [13,14,15,16,17,18,19,20], which reduces proteinuria and mitigates the decline in chronic eGFR slope over time, thereby decreasing the risk of CKD progression and ESKD [13,14,15,16]. Dipeptidyl peptidase-4 inhibitor (DPP4i) is a widely used drug for T2D that promotes insulin secretion and lowers glucagon levels by inhibiting the dipeptidyl peptidase-4 enzyme. Comparative real-world studies showed that SGLT2i treatment was associated with a lower risk of ESKD as well as a mitigation in chronic eGFR slope compared to DPP4i treatment [21,22,23,24,25]. However, it is unclear whether SGLT2i or DPP4i therapy can influence the eGFR variability in individuals with T2D at risk. This study aims to explore how SGLT2i and DPP4i therapy affect different changes in eGFR variability and related adverse kidney events in people with T2D, with varying baseline levels of eGFR variability, in a real-world clinical setting.

## 2. Patients, Materials, and Methods

### 2.1. Database

The present study utilized electronic medical data from Chang Gung Memorial Hospital’s (CGMH), the largest healthcare system in Taiwan. The CGMH system comprises a network of 10 hospital branches (3 medical centers, 5 regional/district hospitals, and 2 municipal hospitals operating under entrustment) located in 8 cities. With a total capacity of 10,050 beds, CGMH serves approximately 280,000 patients annually [26]. The Chang Gung Medical Foundation’s Institutional Review Board reviewed and approved the present study. The findings and interpretations presented herein do not reflect the position of CGMH.

### 2.2. Study Design

**Figure 1:**
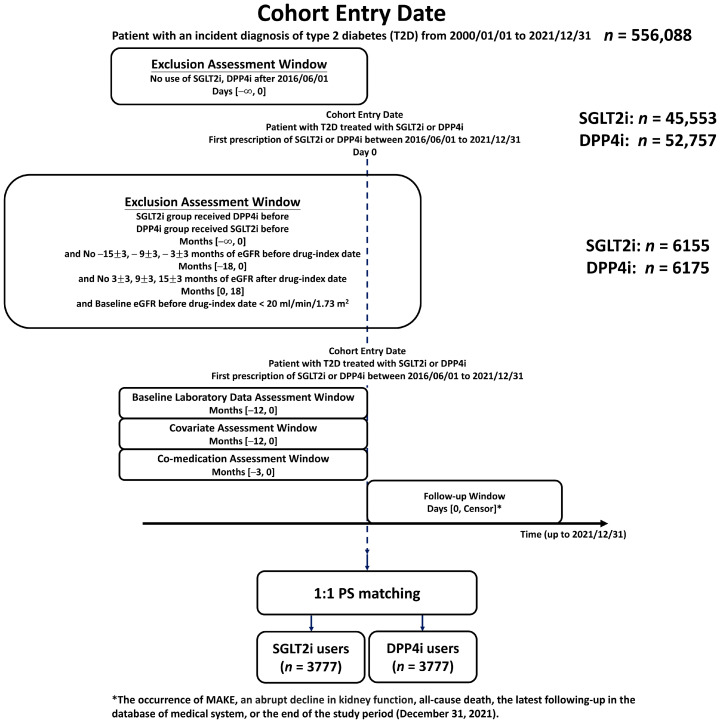
Study design and enrollment of people with type 2 diabetes (T2D) receiving sodium–glucose cotransporter 2 inhibitors (SGLT2is) and dipeptidyl peptidase-4 inhibitors (DPP4is). A total of 6155 and 6175 people with T2D receiving SGLT2i and DPP4i therapy from 1 June 2016, to 31 December 2021, who had at least one eGFR measurement available at −15 ± 3, −9 ± 3, and −3 ± 3 months prior to the drug-index date, as well as follow-up eGFR measurements at 3 ± 3, 9 ± 3, and 15 ± 3 months afterward, were enrolled in the study. There were 3777 paired cohorts of SGLT2i versus DPP4i after propensity score matching (PSM). Abbreviations: DPP4i = dipeptidyl peptidase-4 inhibitor; eGFR = estimated glomerular filtration rate; MAKE = major adverse renal event; PSM = propensity score matching; SGLT2i = sodium–glucose cotransporter 2 inhibitor; T2D = type 2 diabetes.

Figure 1 shows the flowchart outlining the study design and patient selection process. Between 1 January 2000 and 31 December 2021, there were a total of 556,088 individuals with an incident diagnosis of T2D enrolled in the present study. Given that the SGLT2i were introduced later in Taiwan (1 May 2016) compared to DPP4i, the drug-index date was defined as the first prescription of either drugs after 1 June 2016, to ensure a direct comparison. A total of 45,553 patients received their first SGLT2i prescriptions—specifically, empagliflozin and dapagliflozin (approved on 1 May 2016), as well as canagliflozin (approved on 1 March 2018)—while a total of 52,757 patients received DPP4i treatment (including sitagliptin, vildagliptin, saxagliptin, linagliptin, or alogliptin) during the same period. According to Taiwan’s National Health Insurance policy, concurrent or combination use of SGLT2is and DPP4is is not reimbursed due to the financial restrictions. Therefore, individuals with prior exposure to both drug classes before the drug-index date were excluded to ensure mutually exclusive comparison groups. Patients were eligible for inclusion with at least one eGFR measurement available at −15 ± 3, −9 ± 3, and −3 ± 3 months prior to the drug-index date, as well as follow-up eGFR measurements at 3 ± 3, 9 ± 3, and 15 ± 3 months afterward. Additionally, only patients with a baseline eGFR > 20 mL/min/1.73 m^2^ were included. Finally, the study cohort consisted of 6155 and 6175 individuals receiving SGLT2i and DPP4i treatment, respectively, each with baseline data of eGFR slope and variability available. All eGFR values were calculated using the CKD-EPI equation (2021) [27].

### 2.3. Study Outcomes

The following clinical events occurred after the drug-index date were reported: (i) major adverse kidney event (MAKE), defined as either a sustained >50% decline in follow-up eGFR or the development of ESKD, defined as an eGFR of <15 mL/min/1.73 m^2^ during the follow-up period, and (ii) an abrupt decline in kidney function, specified as a doubling of serum creatinine between two subsequent eGFR measurements during the follow-up period [28]. The present analysis only considered the study events that occurred three months later after the drug-index date. Patients were followed up until the occurrence of any study outcome, all-cause death, the latest recorded follow-up date in the CGMH medical system, or the end of the study period (31 December 2021), whichever came first.

### 2.4. Covariates

We identified baseline characteristics from any claims record containing specified diagnoses or medication codes documented prior to the drug-index date. A prescription medication history was determined based on prescriptions filled at least once within the three months preceding the drug-index date. Baseline laboratory data, as summarized in Table 1, were obtained from measurements conducted within one year prior to the drug-index date. We adopted the laboratory measurement closest to the drug-index date for those with multiple laboratory measurements within one year preceding the drug-index date.

### 2.5. Statistical Analysis

Continuous variables were presented as means with standard deviations (SD), while categorical variables were summarized as proportions. We conducted propensity score matching (PSM) in a 1:1 ratio using logistic regression to enable a direct comparison between the SGLT2i and DPP4i groups. We applied the nearest-neighbor algorithm without replacement, incorporating all baseline characteristics listed in Table 1. This matching approach aimed to balance the treatment groups with respect to demographics, comorbidities, laboratory data, and medication profiles [29,30]. We used the absolute standardized mean difference (ASMD) to assess the covariate balance between the matched study groups at the drug-index date. Unlike conventional hypothesis testing, ASMD quantifies the degree of balance between covariates, focusing on the sample rather than inference to the population. An ASMD ≤ 0.1 was considered indicative of adequate covariate balance [31]. The crude incidence rate was calculated by dividing the total number of observed outcomes by the corresponding person-years at risk. The Cox proportional-hazard regression model was employed to compare the risk of adverse events associated with SGLT2i or DPP4i therapy. To evaluate the chronic eGFR slope, a linear regression model was applied to all eGFR values collected from 18 months prior to the drug index date until the last available measurement before treatment and from 3 months post-treatment initiation to the end of follow-up [32,33]. The eGFR variability before and after treatment was assessed using both the coefficient of variation (COV) and the standard deviation (SD) of three eGFR measurements per individual. The eGFR variability before treatment was determined using values from 15 ± 3, 9 ± 3, and 3 ± 3 months before staring the drug, while the eGFR variability after treatment was calculated from measurements taken 3 ± 3, 9 ± 3, and 15 ± 3 months after starting the drug treatment. The COV value was calculated using the following formula: SD of three eGFR values/mean of three eGFR values. When multiple values were available within a designated time window, the measurement closest to the target month (i.e., −15, −9, −3, +3, +9, and +15 months) was selected. The treatment effect of SGLT2i versus DPP4i therapy on baseline eGFR variability was further analyzed using multivariable linear regression, adjusting for age, sex, diabetes duration, baseline comorbidities, body weight, laboratory values at baseline, pre-treatment eGFR slope, systolic blood pressure, heart rate, and use of cardiovascular and glucose-lowering medications, listed in Table 1. The treatment effect of SGLT2i versus DPP4i therapy on adverse kidney outcomes over the pre-treatment eGFR variability as a continuous variable was modeled using a restricted cubic spline with four knots. Missing baseline data were imputed using the expectation–maximization algorithm, an iterative technique that replaces missing values based on the empirical mean and variance–covariance structure of the observed data [34,35]. All statistical analyses were performed using SAS 9.4 (SAS Institute, Cary, NC, USA), SPSS 26.0 (IBM Corp., Armonk, NY, USA), or R Statistics 4.3.3 (R Foundation for Statistical Computing, Vienna, Austria). A two-sided *p* value of <0.05 was considered statistically significant.

## 3. Results

### 3.1. The Baseline Characteristics of Patients Receiving SGLT2i and DPP4i Treatment

A total of 6155 and 6175 patients receiving SGLT2i and DPP4i therapy, respectively, were eligible for inclusion in the present study. Table 1 presents the baseline characteristics of the two treatment groups before and after PSM. Prior to matching, there were substantial differences observed between the groups, with most ASMD > 0.10. Following PSM, 3077 matched pairs of patients receiving SGLT2i and DPP4i therapy were identified. Baseline characteristics were well-balanced in the matched cohort, with all ASMD < 0.10.

### 3.2. Pre-Treatment and Post-Treatment eGFR Slope and Variability in Patients Receiving SGLT2i and DPP4i Treatment

**Figure 2:**
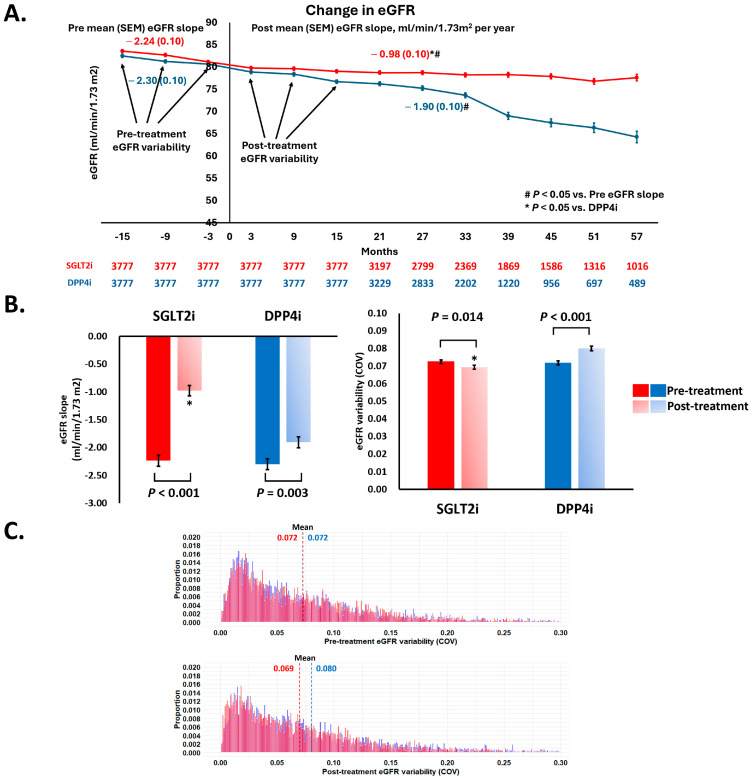
Changes in the mean (SEM) eGFR trajectories over time (**A**), changes in the eGFR decline slope, eGFR variability assessed by COV (**B**), and changes in number proportion with different eGFR variability (**C**) before and after drug-index date in patients receiving SGLT2i and DPP4i therapy. COV = coefficient of variation; SEM = standard error of the mean. COV value is calculated using the following formula: SD of three eGFR values/mean of three eGFR values). Other abbreviations are as in Figure 1. * *p* < 0.05 vs. DPPi.

SGLT2i and DPP4i treatments reduced the mean (SEM) decline in eGFR from −2.24 (0.10) to −0.98 (0.10) (*p* < 0.001) and from −2.30 (0.10) to −1.90 (0.10) mL/min/1.73 m^2^ per year (*p* = 0.003). Patients receiving SGLT2i treatment were associated with a slower eGFR annual decline compared to those receiving DPP4i treatment (−0.98 (0.10) versus −1.90 (0.10) mL/min/1.73 m^2^ per year; *p* < 0.001). It is noted that SGLT2i treatment was associated with an improvement in eGFR variability estimated by either COV (from 0.072 (0.001) to 0.069 (0.001); *p* = 0.014) or SD (from 5.34 (0.07) to 4.82 (0.07); *p* < 0.001). Conversely, DPP4i treatment was not associated with stabilization of eGFR variability estimated by either COV (from 0.072 (0.001) to 0.080 (0.001); *p* < 0.001) or SD (from 5.06 (0.07) to 5.22 (0.07); *p* = 0.082) (Figure 2 and Supplemental Figure S1).

The decrease in eGFR variability after SGLT2i treatment, adjusted for DPP4i, was −0.011 (95% confidence interval (CI) −0.015 to −0.008; *p* < 0.001) when measured by COV and −0.668 mL/min/1.73 m^2^ (95% CI −0.907 to −0.430; *p* < 0.001) when measured by SD. This reduction in eGFR variability for SGLT2i in relation to DPP4i was similar across most groups, including older patients, female in gender, those with a higher baseline glycated hemoglobin (HbA1c) > 8.0%, those with baseline albuminuria, patients on a RAAS inhibitor, and those on any diuretics. However, there was evidence of a greater reduction in eGFR variability estimated by COV in patients with baseline eGFR < 60 mL/min/1.73 m^2^ and rapid pre-treatment eGFR decline > 3 mL/min/1.73 m^2^ per year (Supplemental Figures S2 and S3).

**Figure 3:**
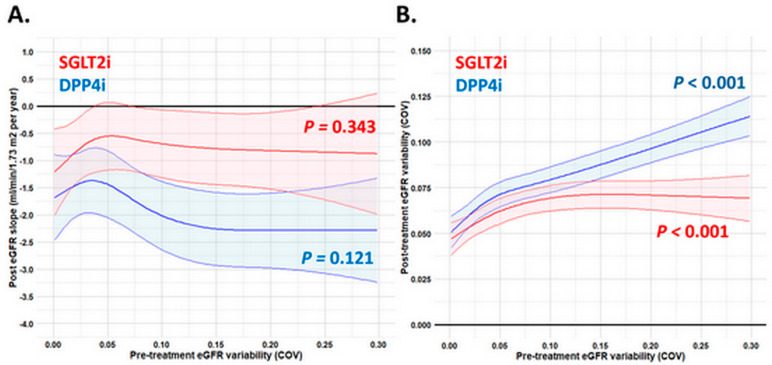
The post-treatment eGFR slope (**A**) and variability (**B**) across the range of pre-treatment eGFR variability (COV) examined as a continuous variable in patients receiving SGLT2i (red) and DPP4i (blue) therapy. The abbreviations are as in Figure 1 and Figure 2. The post-treatment eGFR slope and variability was adjusted for age, gender, duration of diabetes, all baseline comorbidities, baseline body weight, HbA1c, baseline eGFR, UACR, lipid profiles, pre-treatment eGFR decline slope, systolic blood pressure, heart rate, all baseline cardiovascular drugs, and anti-hyperglycemic agents in Table 1.

Overall, SGLT2i treatment was associated with a slower annual decline in post-treatment eGFR compared to DPP4i treatment, regardless of the range of pre-treatment eGFR variability examined as a continuous variable (Figure 3A and Supplemental Figure S4A). Regardless of whether variability is measured using COV or SD, post-treatment eGFR variability tends to increase with higher pre-treatment variability, either for the SGLT2i and DPP4i group. Both study groups show an increase, but the rise in post-treatment eGFR variability is steeper for the DPP4i therapy than for the SGLT2i therapy, especially when pre-treatment eGFR variability is high. These findings may suggest that, in individuals with greater pre-treatment eGFR variability, DPP4i therapy is associated with a more pronounced increase in the post-treatment eGFR variability compared to SGLT2i therapy (Figure 3B and Supplemental Figure S4B).

### 3.3. Risk of Adverse Kidney Outcomes with Different Pre-Treatment eGFR Variability in Patients Receiving SGLT2i vs. DPP4i Treatment

**Figure 4:**
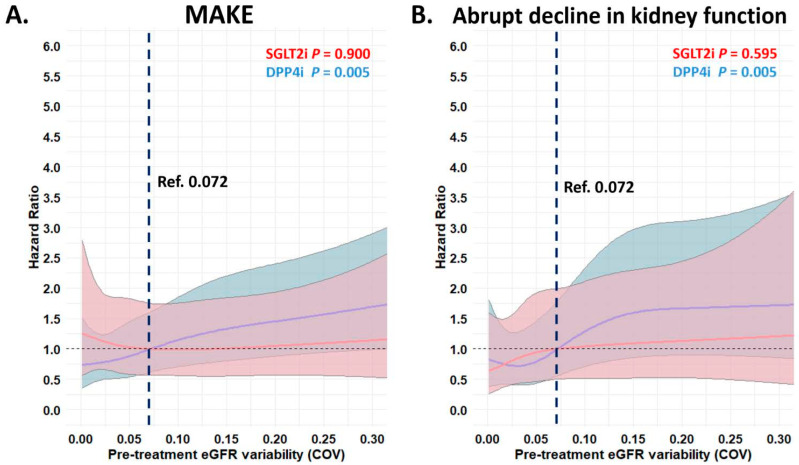
Risk of adverse kidney outcomes of MAKE (**A**) and abrupt decline in kidney function (**B**) for the paired study cohorts receiving SGTL2i (red) or DPP4i (blue) after PSM across the range of different pre-treatment eGFR variability (COV) examined as a continuous variable. MAKE = major adverse kidney event. Other abbreviations are as in Figure 1 and Figure 2. The adjusted factor for clinical outcomes is as in Figure 3.

**Figure 5:**
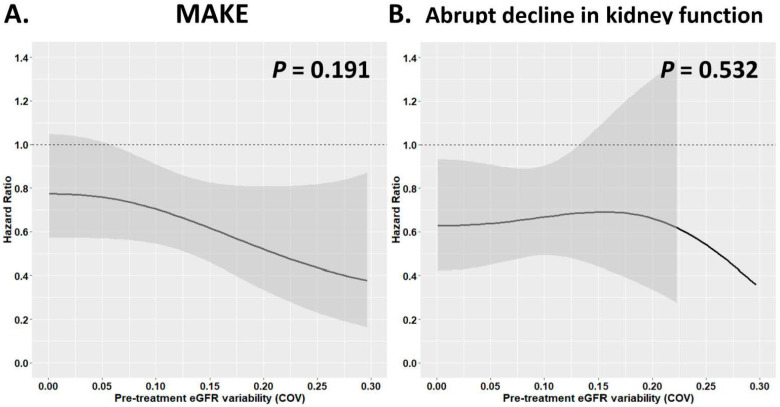
The treatment effect of MAKE (**A**) and abrupt decline in kidney function (**B**) for SGTL2i versus DPP4i after PSM across the range of pre-treatment eGFR variability (COV) examined as a continuous variable. The abbreviations are as in Figure 1, Figure 2 and Figure 4. The adjusted factor for clinical outcomes is as in Figure 3.

Modeling the different pre-treatment eGFR variability at baseline as a continuous variable with a restricted cubic splines model, we found that a greater eGFR variability either estimated by COV or SD before starting the index drug was independently associated with a higher risk of MAKE and an abrupt decline in kidney function in patients receiving DPP4i therapy (*p* both < 0.01), but this was not the case for those on SGLT2i therapy (Figure 4 and Supplemental Figure S5). Overall, the benefit of SGLT2i compared with DPP4i treatment was consistent for the outcome of MAKE and an abrupt decline in kidney function across the range of pre-treatment eGFR variability examined as a continuous variable. There was a statistically significant interaction when comparing associations between the increase in the pre-treatment eGFR variability estimated by SD and the treatment effect on the MAKE for SGLT2i compared to DPP4i treatment (*p*-interaction = 0.014) (Figure 5 and Supplemental Figure S6).

## 4. Discussion

In the present study, we looked at 3777 matched individuals with T2D who were treated with SGLT2i and DPP4i treatment to evaluate how their eGFR variability changed before and after drug therapy, as well as several consequent adverse kidney outcomes with interest. Overall, SGLT2i treatment was associated with an improvement in eGFR variability, while DPP4i treatment was not. Patients who had higher eGFR variability before starting drug therapy showed a greater reduction in eGFR variability with SGLT2i therapy compared to DPP4i therapy, especially if they already had CKD at baseline or a rapid decline in eGFR slope (>3 mL/min/1.73 m^2^ per year) before treatment. A greater pre-treatment eGFR variability was independently associated with a higher risk of major adverse kidney events (MAKEs) and an abrupt decline in kidney function in patients receiving DPP4i therapy but not in those receiving SGLT2i therapy. Overall, the advantage of SGLT2i compared to DPP4i treatment was consistent for the risk of MAKE and an abrupt decline in kidney function, regardless of the range of pre-treatment eGFR variability examined as a continuous measure. Of note, there was a greater risk reduction in the risk of MAKE for SGLT2i compared to DPP4i therapy with a greater pre-treatment eGFR variability as estimated by SD (*p* interaction = 0.014).

Rapid eGFR decline has significant clinical implications, which markedly increase the risk of kidney disease progression and cardiovascular complications [3,36]. Of note, a rapid decline in eGFR, defined as an annual eGFR decline of ≥5 mL/min/1.73 m^2^ (or other studies have utilized a threshold of a decline of ≥3 mL/min/1.73 m^2^ per year to define rapid eGFR decline [37,38]), is a strong predictor of adverse cardiovascular outcomes and ESKD [32,39]. Additionally, eGFR is often assessed repeatedly to monitor kidney disease progression in clinical practice, and thus, eGFR variability of varying degrees is commonly observed. Previous studies have shown that greater eGFR variability was associated with new or worsening kidney disease and a higher risk of dialysis, major adverse cardiovascular events, and all-cause mortality in patients with T2D, CKD, or heart failure, independent of the absolute eGFR value or chronic slope [6,7,8,9,10,11,12].

The underlying mechanisms contributing to a greater eGFR variability are multifactorial, reflecting the impairment in both intrinsic kidney autoregulation and systemic physiological instability. Tubuloglomerular feedback (TGF), a key autoregulatory mechanism, is often blunted in pathological conditions like obesity, diabetes, and CKD, resulting in unstable glomerular filtration rate [40]. Episodes of dehydration, acute illness, infections, or heart failure exacerbations can transiently alter kidney perfusion and lead to abrupt changes in eGFR [41]. Several medications, including RAAS inhibitors, calcium channel blockers, NSAIDs, and diuretics, may also contribute to variability by affecting intrarenal hemodynamics or volume status [42]. In addition, laboratory-related factors—such as diet, muscle mass, creatinine assay variation, circadian variations, hydration status, and physical activity—introduce biological noise that can further affect serum creatine and eGFR calculations [43]. Vascular and endothelial dysfunction, commonly present in aging, diabetes, hypertension, and CKD, impair microvascular responsiveness, thereby amplifying eGFR instability [44].

So far, the post hoc analyses of major randomized controlled trials and real-world studies of SGLT2i have mainly focused on the eGFR slope and several composite kidney outcomes, such as key renal outcomes, with limited exploration of eGFR variability (e.g., SD or COV) as a pre-specified kidney outcome, or how they affect long-term kidney outcome in patients taking SGLT2i, placebo, or an active comparator. While the eGFR decline slope is well-established as a kidney endpoint in SGLT2i trials, eGFR variability remains an emerging concept. Our study showed that SGLT2i therapy was associated with a blunted increase in the eGFR variability after treatment compared to DPP4i therapy in people who had higher eGFR variability before treatment started. We also found that a greater pre-treatment eGFR variability was independently associated with a higher risk of major adverse kidney events in patients taking DPP4i therapy, but this was not the case for those on SGLT2i therapy. This finding suggests that SGLT2i intervention might help reduce the negative effects usually linked to higher eGFR variability prior to drug therapy.

SGLT2is specifically restore TGF by augmenting sodium delivery to the macula densa, resulting in afferent arteriolar vasoconstriction and decreased intraglomerular pressure [45]. This process directly reduces the hyperfiltration and hemodynamic stress that cause fluctuation in eGFR for patients with diabetes, CKD, or heart failure. Additionally, SGLT2i reduces inflammation, oxidative stress, and fibrosis, thus enhancing kidney microvascular stability and maintaining structural integrity [46]. These effects are believed to diminish both the magnitude and clinical implications of eGFR variability. Consequently, patients with greater pre-treatment eGFR variability may not encounter a higher incidence of adverse kidney outcomes following SGLT2i treatment, as this variability typically indicates subclinical renal susceptibility. This finding highlights that SGLT2i can not only mitigate the decline in long-term eGFR slope but also lessen the impact of how much eGFR varies before starting the treatment, showing their important role for diabetic patients who are at varied risk for DKD progression.

Several real-world studies indicated that SGLT2i treatment ameliorated the chronic eGFR decline slope compared to DPP4i treatment in patients with T2D, with or without evidence of cardiovascular or kidney disease [21,22,23]. However, data specifically examining eGFR variability are also lacking. Our present study showed greater eGFR variability despite the relatively stable eGFR decline slope in those receiving DPP4i treatment, suggesting that these two kidney indicators may reflect distinct pathophysiological processes. The eGFR decline slope signifies a sustained deterioration in kidney function, while the eGFR variability indicates short-term variations that may be influenced by temporary hemodynamic changes, pharmacological factors, and systemic instability. DPP4is therapy is generally considered to have neutral effects on eGFR decline slope compared to placebo, with some studies indicating a reduction in the progression of albuminuria in the early stages of DKD [47]. Both SGLT2i and GLP-1 receptor agonists exert their primary natriuretic action in the proximal tubule. In contrast, the primary natriuretic effect of DPP4i is located in the distal tubule (beyond the macula densa), thereby leaving TGF, afferent resistance, and intraglomerular pressure unaltered [48]. The modest effect of DPP4i to reduce albuminuria may reflect an action to minimize hyperglycemia-induced increases in podocyte protein permeability, rather than a benefit on nephropathy [49]. Although some animal studies showed that DPP4i may suppress kidney oxidative stress and fibrosis through the SDF-1α/CXCR4-depdendent signaling pathway [50], the proinflammatory effects of SDF-1 can also exacerbate the progression of kidney disease [51]. Consequently, DPP4i may be ineffective in mitigating fluctuations in glomerular pressure, leaving patients vulnerable to transient alterations in filtration due to variations in volume status, blood pressure changes, or coexisting medical conditions like CKD or heart failure. Consequently, despite a stable overall decline in kidney function, patients receiving DPP4i may demonstrate increased eGFR variability, potentially indicating persistent subclinical kidney stress or an inability to accommodate short-term fluctuations in kidney function.

## 5. Study Limitations

This study has several limitations. It is a retrospective and observational study. Several baseline characteristics varied significantly across the pre-matched study cohorts. The PSM created a comparable cohort by balancing baseline characteristics, but at the expense of a smaller representation than that of the actual study population. This study adjusted for a wide range of clinical factors at baseline. Due to incomplete data, we were unable to adjust for other key factors like smoking, alcohol, physical activity, or body composition. We used serial serum creatinine measurements to determine the eGFR slopes and variability rather than cystatin C measurements, which means that the eGFR values we obtained might be affected by changes in muscle mass or body composition. Our study only enrolled people with at least three checkups before and after treatment to calculate eGFR variability. Therefore, we cannot rule out the possibility of selection bias. We calculated the COV and SD of eGFR by using only three eGFR data points, each approximately six months apart [7], so the prognostic value of shorter or longer-term eGFR variability is unknown. Additionally, since this study focused on an Asian population with diabetes, concerns regarding generalizability also exist.

## 6. Conclusions

SGLT2i treatment was associated with an improvement in eGFR variability and a lower risk of major adverse kidney outcomes compared to DPP4i treatment, with a greater treatment benefit in patients with higher pre-treatment eGFR variability. SGLT2i therapy reduced eGFR variability more than DPP4i therapy in high-risk populations, including those with greater eGFR variability, the presence of CKD, or a rapid pre-treatment eGFR decline at baseline.

## Figures and Tables

**Table 1:** Clinical characteristics of the people with type 2 diabetes (T2D) treated with SGLT2i and DPP4i before and after propensity score matching (PSM).

	Before PSM			After PSM		
	SGLT2i (*n* = 6155)	DPP4i (*n* = 6175)	ASMD	SGLT2i (*n* = 3777)	DPP4i (*n* = 3777)	ASMD
**Baseline characteristics**						
**Diabetes duration**	8.2 ± 5.3	8.0 ± 5.7	0.035	8.1 ± 5.3	8.0 ± 5.7	0.011
**Age (mean ± SD)**	62.4 ± 11.4	68.5 ± 11.4	0.537	65.0 ± 10.6	65.6 ± 11.3	0.048
**Male**	3732 (61)	3280 (53)	0.152	2124 (56)	2116 (56)	0.004
**Ischemic heart etiology**	683 (11)	397 (6)	0.166	312 (8)	299 (8)	0.013
**Cerebral vascular accidents**	100 (2)	161 (3)	0.068	80 (2)	83 (2)	0.005
**Congestive heart failure**	306 (5)	226 (4)	0.065	145 (4)	144 (4)	0.001
**Chronic lung disease**	198 (3)	262 (4)	0.054	136 (4)	138 (4)	0.003
**Chronic liver disease**	2112 (34)	2075 (34)	0.015	1277 (34)	1236 (33)	0.023
**Peripheral artery disease**	58 (1)	73 (1)	0.023	39 (1)	45 (1)	0.015
**Gout**	865 (14)	972 (16)	0.047	529 (14)	532 (14)	0.002
**Malignancy**	671 (11)	1216 (20)	0.246	518 (14)	553 (15)	0.027
**Baseline vital signs**						
**Baseline body weight (KG)**	74.9 ± 15.3	67.4 ± 12.9	0.532	70.8 ± 12.7	70.2 ± 13.4	0.044
**Baseline SBP (mmHg)**	139.7 ± 19.4	138.5 ± 19.8	0.058	139.1 ± 19.1	139.2 ± 19.4	0.002
**Baseline DBP (mmHg)**	78.1 ± 11.9	76.2 ± 12.4	0.163	77.1 ± 11.4	77.2 ± 12.0	0.007
**Baseline heart rate (bpm)**	83.8 ± 13.6	82.8 ± 13.9	0.073	83.0 ± 13.5	83.2 ± 13.9	0.014
**Baseline laboratory data**						
**Pre-treatment eGFR slope** **(mL/min/1.73 m^2^/year) (med, IQR)**	−1.37 (−4.34, 1.28)	−2.18 (−5.78, 0.22)	0.225	−1.70 (−4.92, 0.95)	−1.78 (−4.72, 0.63)	0.010
**Pre-treatment eGFR COV** **(med, IQR)**	0.050 (0.022, 0.096)	0.060 (0.027, 0.110)	0.157	0.055 (0.025, 0.101)	0.052 (0.023, 0.096)	0.009
**Pre-treatment eGFR SD** **(mL/min/1.73 m^2^/year) (med, IQR)**	4.04 (2.15, 7.18)	4.12 (2.18, 7.23)	0.011	4.17 (2.19, 7.23)	3.95 (2.03, 6.90)	0.065
**Baseline eGFR** **(mL/min/1.73 m^2^/year)**	85.4 ± 21.3	75.0 ± 25.0	0.447	81.2 ± 21.1	80.7 ± 23.9	0.022
**Baseline urine albumin-to-creatinine ratio** **(mg/g) (med, IQR)**	63.9 (12.0, 275.1)	72.0 (12.0, 384.0)	0.065	61.7 (11.3, 282.0)	65.0 (11.0, 376.0)	0.008
**Baseline HbA1c (%)**	8.3 ± 1.5	7.6 ± 1.4	0.478	8.0 ± 1.3	7.9 ± 1.6	0.047
**Baseline ALT (U/L)**	35.8 ± 33.6	31.6 ± 33.5	0.125	33.4 ± 29.7	33.5 ± 30.8	0.003
**Baseline triglycerides (mg/dL)**	184.8 ± 219.8	156.0 ± 122.6	0.162	163.0 ± 131.3	159.0 ± 127.4	0.031
**Baseline LDL (mg/dL)**	91.7 ± 29.1	97.3 ± 61.2	0.116	92.8 ± 29.3	92.6 ± 35.8	0.007
**Baseline HDL (mg/dL)**	44.3 ± 11.3	46.0 ± 12.3	0.145	45.3 ± 11.6	45.5 ± 12.2	0.014
**Baseline medications**						
**Use of anti-platelet agent**	2009 (33)	1763 (29)	0.089	1144 (30)	1129 (30)	0.009
**Use of statin**	3808 (62)	3484 (56)	0.111	2281 (60)	2280 (60)	0.001
**Use of CCB**	1136 (18)	1489 (24)	0.139	770 (20)	763 (20)	0.005
**Use of beta-blocker**	2302 (37)	1801 (29)	0.175	1247 (33)	1222 (32)	0.014
**Use of RAAS inhibitor**	4031 (65)	3674 (59)	0.124	2379 (63)	2349 (62)	0.016
**Use of loop diuretics**	461 (7)	667 (11)	0.115	308 (8)	316 (8)	0.008
**Use of thiazide**	1114 (18)	937 (15)	0.079	631 (17)	608 (16)	0.016
**Use of MRA**	232 (4)	244 (4)	0.009	139 (4)	131 (3)	0.011
**Use of vasodilator**	333 (5)	294 (5)	0.030	182 (5)	178 (5)	0.005
**Use of NSAIDs**	776 (13)	1051 (17)	0.124	554 (15)	542 (14)	0.009
**Use of UA lowering agent**	656 (11)	873 (14)	0.106	428 (11)	466 (12)	0.031
**Use of anti-diabetic agent**						
** ** **Metformin**	5488 (89)	5051 (82)	0.210	3305 (88)	3285 (87)	0.016
** ** **SU**	3211 (52)	2564 (42)	0.215	1785 (47)	1760 (47)	0.013
** ** **Glinide**	176 (3)	340 (6)	0.132	140 (4)	135 (4)	0.007
** ** **Glitazone**	1133 (18)	373 (6)	0.384	404 (11)	352 (9)	0.046
** ** **Acarbose**	910 (15)	719 (12)	0.093	491 (13)	498 (13)	0.005
** ** **Insulin**	942 (15)	703 (11)	0.115	488 (13)	459 (12)	0.023

ASMD, absolute standardized mean difference; ALT, alanine aminotransferase; CCB, calcium channel blocker; COV, coefficient of variation; DBP, diastolic blood pressure; DPP4i, dipeptidyl peptidase-4 inhibitor; eGFR, estimated glomerular filtration rate; HBA1c, hemoglobin A1c; HDL, high density lipoprotein; LDL, low density lipoprotein; MRA, mineralocorticoid receptor antagonist; NSAIDs, non-steroidal anti-inflammatory drugs; PSM, propensity score matching; RAAS, renin–angiotensin–aldosterone system; SBP, systolic blood pressure; SD, standard deviation; SGLT2i, sodium–glucose co-transporter-2 inhibitor; SU, sulfonylurea; T2D, type 2 diabetes, UA, uric acid. Data are expressed as the mean ± standard deviation (SD), (med, IQR)**,** or as percentage. The eGFR was calculated using the CKD-EPI 2021 equation.

## Data Availability

The datasets used in this study are only available in the Chang Gung Medical Data Center, Taiwan. The programs (codes) used in this study are available from the corresponding author on reasonable request.

## Supplementary Materials

The following supporting information can be downloaded at: https://www.mdpi.com/article/10.3390/pharmaceutics17111370/s1. Supporting Results including: Figure S1. Changes of the eGFR variability assessed by SD and changes in number proportion with different eGFR variability before and after drug-index date in patients receiving SGLT2i and DPP4i therapy. Figure S2. The DPP4i-corrected reduction in post-treatment eGFR variability (COV) from baseline for SGLT2i treatment. Figure S3. The DPP4i-corrected reduction in post-treatment eGFR variability (SD) from baseline for SGLT2i treatment. Figure S4. The post-treatment eGFR slope (A) and variability (B) across the range of pre-treatment eGFR variability (SD) examined as a continuous variable in patients receiving SGLT2i and DPP4i therapy. Figure S5. Risk of adverse kidney outcomes for the paired study cohorts receiving SGTL2i or DPP4i after PSM across the range of different pre treatment eGFR variability (SD) examined as a continuous variable. Figure S6. The treatment effect for SGTL2i versus DPP4i after PSM across the range of pre-treatment eGFR variability (SD) examined as a continuous variable.

## Abbreviations

**ALT**	alanine aminotransferase
**BMI**	body mass index
**BP**	blood pressure
**BW**	body weight
**CI**	confidence interval
**CKD**	chronic kidney disease
**COV**	coefficient of variation
**DPP4i**	dipeptidyl peptidase-4 inhibitor
**eGFR**	estimated glomerular filtration rate
**ESKD**	end-stage kidney disease
**HbA1c**	glycated hemoglobin
**HDL**	high-density lipoprotein
**HR**	hazard ratio
**LDL**	low-density lipoprotein
**MAKE**	major adverse kidney event
**NSAIDs**	nonsteroidal anti-inflammatory drugs
**RAAS**	renin–angiotensin system
**SGLT2i**	sodium–glucose cotransporter-2 inhibitor
**SD**	standard deviation
**T2D**	type 2 diabetes
**UACR**	urine albumin-to-creatinine ratio
