# Supplementary material for: Estimated Glomerular Filtration Rate Variability in Patients with Diabetes Receiving SGLT2 Inhibitors Versus DPP4 Inhibitors
Source: Pharmaceutics. 2025 Oct 23;17(11):1370. doi: 10.3390/pharmaceutics17111370 (PMC12655220; doi:10.3390/pharmaceutics17111370)

Supplemental Material

Supplemental Figure S1. Changes of the eGFR variability assessed by SD and changes in number proportion with different eGFR variability before and after drug-index date in patients receiving SGLT2i and DPP4i therapy

Abbreviations:

COV = coefficient of variation; DPP4i = dipeptidyl peptidase-4 inhibitor; eGFR = estimated glomerular filtration rate; SD = standard deviation; SGLT2i = sodium glucose cotransporter 2 inhibitor

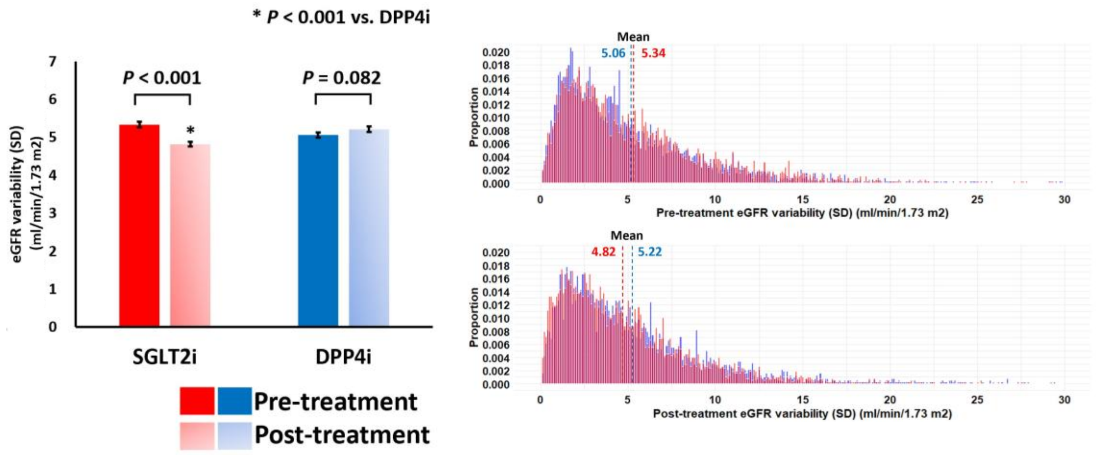

Supplemental Figure S2. The DPP4i-corrected reduction in post-treatment eGFR variability (COV) from baseline for SGLT2i treatment

Abbreviations:

COV = coefficient of variation; DPP4i = dipeptidyl peptidase-4 inhibitor; eGFR = estimated glomerular filtration rate; HbA1c = glycated hemoglobin; RAASi = renin–angiotensin system inhibitor; SD = standard deviation; SGLT2i = sodium glucose cotransporter 2 inhibitor

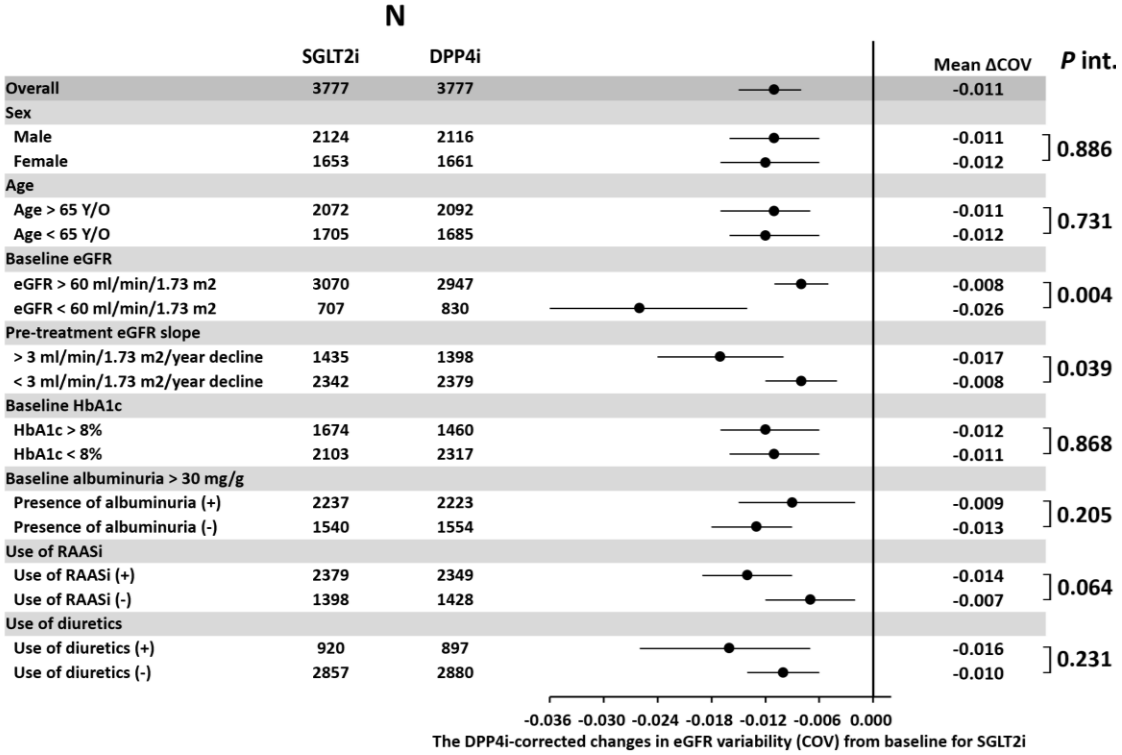

Supplemental Figure S3. The DPP4i-corrected reduction in post-treatment eGFR variability (SD) from baseline for SGLT2i treatment

Abbreviations:

COV = coefficient of variation; DPP4i = dipeptidyl peptidase-4 inhibitor; eGFR = estimated glomerular filtration rate; HbA1c = glycated hemoglobin; RAASi = renin–angiotensin system inhibitor; SD = standard deviation; SGLT2i = sodium glucose cotransporter 2 inhibitor

	N		Mean ΔSD (ml/min/1.73 m2)	*P* int.
	SGLT2i	DPP4i		
Overall	3777	3777	-0.668	
Sex				
Male	2124	2116	-0.590	0.470
Female	1653	1661	-0.769	
Age				
Age > 65 Y/O	2072	2092	-0.676	0.934
Age < 65 Y/O	1705	1685	-0.656	
Baseline eGFR				
eGFR > 60 ml/min/1.73 m2	3070	2947	-0.614	0.157
eGFR < 60 ml/min/1.73 m2	707	830	-1.060	
Pre-treatment eGFR slope				
> 3 ml/min/1.73 m2/year decline	1435	1398	-0.831	0.287
< 3 ml/min/1.73 m2/year decline	2342	2379	-0.551	
Baseline HbA1c				
HbA1c > 8%	1674	1460	-0.459	0.156
HbA1c < 8%	2103	2317	-0.818	
Baseline albuminuria > 30 mg/g				
Presence of albuminuria (+)	2237	2223	-0.598	0.481
Presence of albuminuria (-)	1540	1554	-0.769	
Use of RAASi				
Use of RAASi (+)	2379	2349	-0.838	0.068
Use of RAASi (-)	1398	1428	-0.387	
Use of diuretics				
Use of diuretics (+)	920	897	-0.667	0.998
Use of diuretics (-)	2857	2880	-0.667	

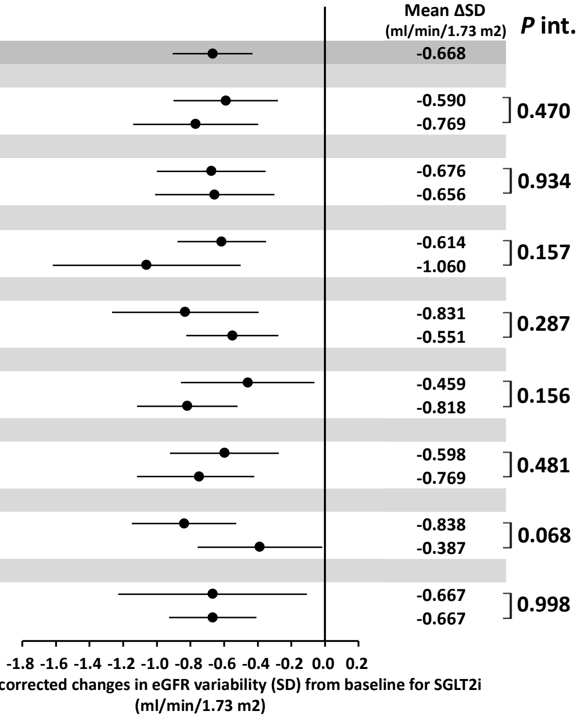

Supplemental Figure S4. The post-treatment eGFR slope (A) and variability (B) across the range of pre-treatment eGFR variability (SD) examined as a continuous variable in patients receiving SGLT2i and DPP4i therapy

Abbreviations:

COV = coefficient of variation; DPP4i = dipeptidyl peptidase-4 inhibitor; eGFR = estimated glomerular filtration rate; SD = standard deviation; SGLT2i = sodium glucose cotransporter 2 inhibitor

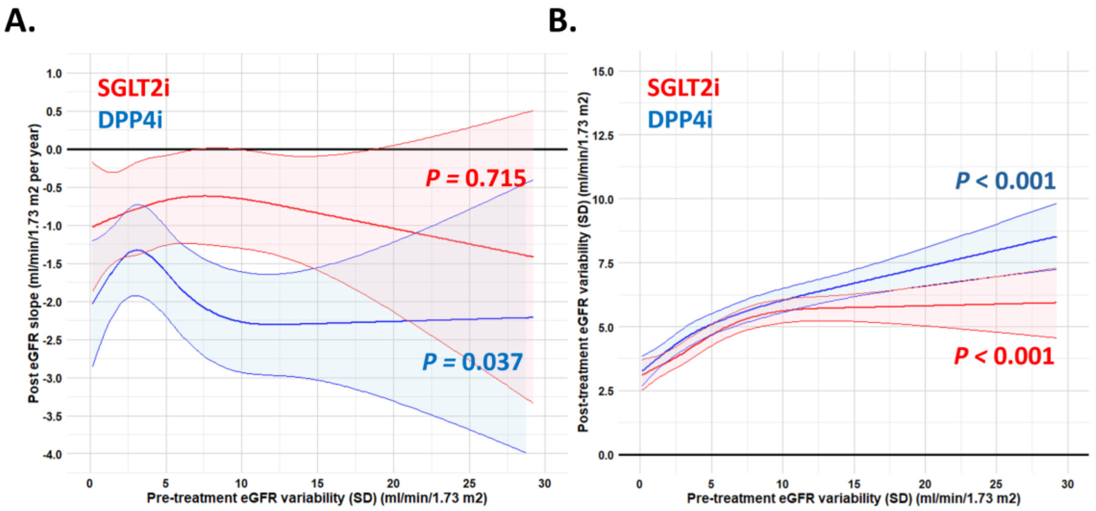

Supplemental Figure S5. Risk of adverse kidney outcomes for the paired study cohorts receiving SGTL2i or DPP4i after PSM across the range of different pre-treatment eGFR variability (SD) examined as a continuous variable

MAKE = major adverse kidney event

Other abbreviations as in Supplemental **Figure S1 to S4**.

The post-treatment eGFR slope and variability was adjusted for age, gender, duration of diabetes, all baseline comorbidities, baseline body weight, HbA1c, baseline eGFR, UACR, lipid profiles, pre-treatment eGFR decline slope, systolic blood pressure, heart rate, all baseline cardiovascular drugs and anti-hyperglycemic agents in **Table 1**.

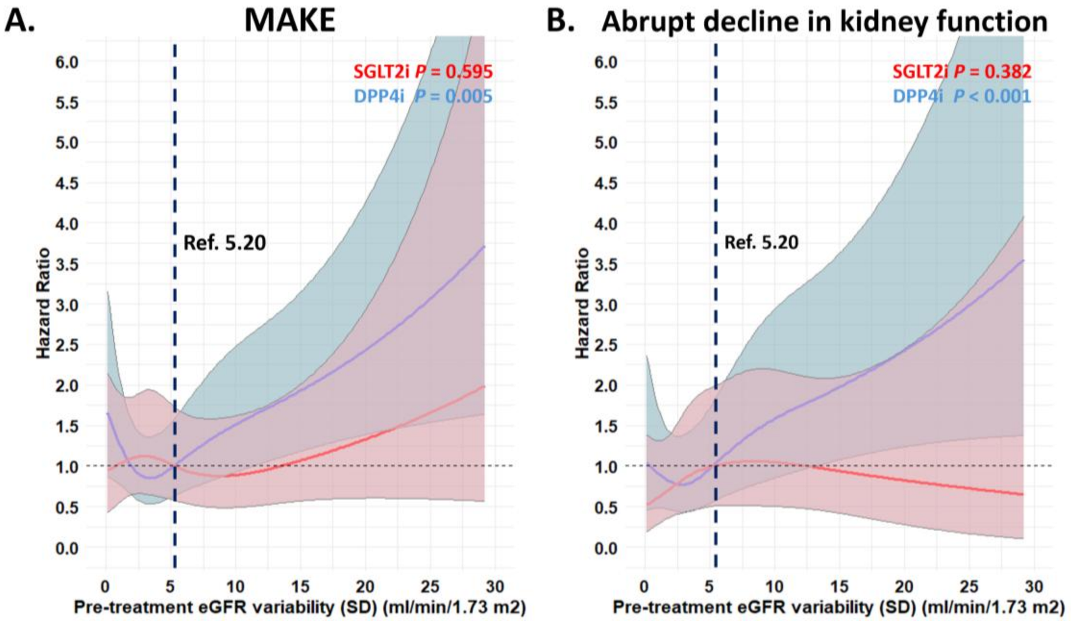

Supplemental Figure S6. The treatment effect for SGTL2i versus DPP4i after PSM across the range of pre-treatment eGFR variability (SD) examined as a continuous variable

MAKE = major adverse kidney event

Other abbreviations as in Supplemental **Figure S1 to 4**.

The post-treatment eGFR slope and variability was adjusted for age, gender, duration of diabetes, all baseline comorbidities, baseline body weight, HbA1c, baseline eGFR, UACR, lipid profiles, pre-treatment eGFR decline slope, systolic blood pressure, heart rate, all baseline cardiovascular drugs and anti-hyperglycemic agents in **Table 1**.

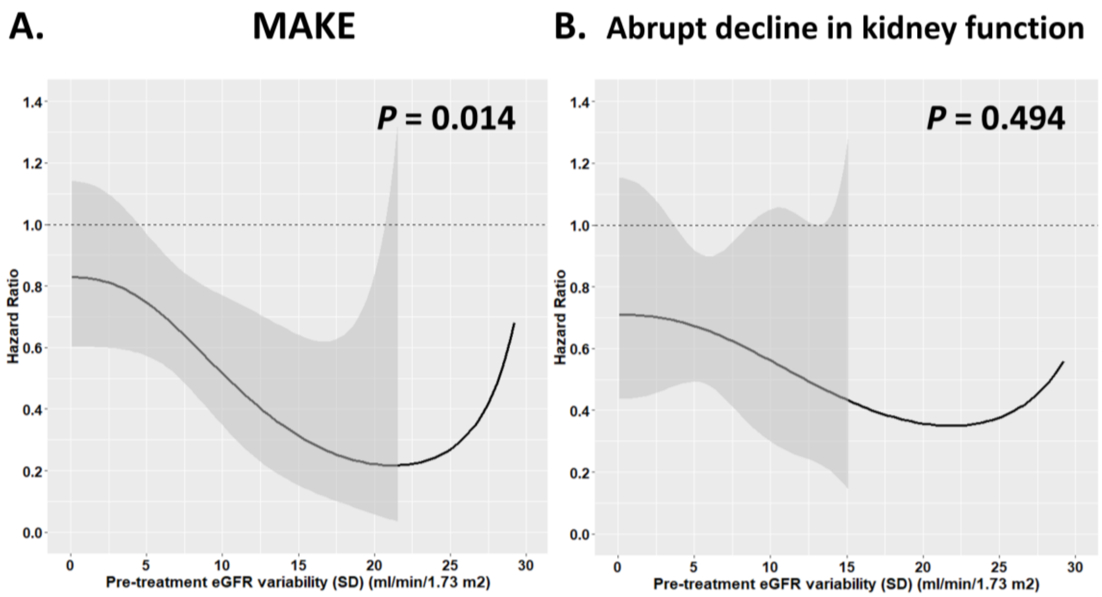

Supplement: Supplementary file 1 [file pharmaceutics-17-01370-s001.zip › pharmaceutics-3915756-supplementary.pdf]
